# Reusability Report: Meta-Learning for Antigen-Specific T-Cell Receptor Binder Identification

**DOI:** 10.21203/rs.3.rs-7456773/v1

**Published:** 2025-09-19

**Authors:** Dong Xu, Fei He, Xianyu Wang

**Affiliations:** University of Missouri - Columbia; University of Missouri - Columbia; University of Missouri-Columbia

## Abstract

Accurate prediction of peptide-T-cell receptor (TCR) binding is vital for immunotherapy, vaccine design, and diagnostics. PanPep, a meta-learning framework, was developed to generalize diverse TCR binder predictions. This study presents a comprehensive and unbiased evaluation of PanPep’s reusability and practical utility. We reproduced its reported performance on original datasets and further benchmarked it against the control tools using both classification metrics and virtual screening enrichment evaluations. Leveraging a newly curated independent dataset, we have demonstrated PanPep’s superior generalization to unseen antigens with few or no known TCR binders. We further extended PanPep to peptide-TCRα and peptide-TCRαβ binding prediction, demonstrating its applicability in more biologically and physiologically relevant contexts. Despite its strengths, PanPep shows limitations in early binder enrichment and reduced robustness to novel TCRs, indicating sensitivity to training data composition and negative sampling strategies. This work establishes a reproducible and extensible benchmarking framework for general peptide-TCR binding prediction and related applications. Overall, our study suggests substantial room for improvement in TCR binder prediction, particularly concerning its practical applicability.

## Introduction

T-cell receptors (TCRs) recognize peptides presented by major histocompatibility complex (MHC) molecules, initiating adaptive immune responses. Identifying peptide-specific TCR binders is crucial for immunotherapy, vaccine design, and diagnostic applications, particularly for targeting neoantigens and novel antigens^1^. Given the vast combinatorial diversity of peptides and TCRs, exhaustive experimental screening is impractical. Computational prediction offers a cost-effective, scalable, and time-efficient alternative, enabling high-throughput *in silico* screening to bridge the gap between sequence diversity and experimental limitations^2^.

Early computational approaches predicted antigen-TCR interactions by grouping TCRs based on sequence similarity, with tools such as GLIPH^3^, GIANA^4^, and DeepTCR^5^. While effective in narrow contexts, these methods were often restricted to antigen types and specific human leukocyte antigen (HLA) alleles with limited generalizability^6^. More recent AI-based tools reformulated the task as a sequence modeling problem, using tokenized amino acids and attention-based architectures to infer peptide-TCR interactions. Representative models include DIpTCR^7^, ERGO^8^, pMTnet^9^, TEIM^10^, and PanPep^11^. Other recent models, such as PISTE^12^, Unifylmmun^13^, and deepAntigen^6^ have expanded the scope to HLA-antigen-TCR multimers.

**Figure 1:**
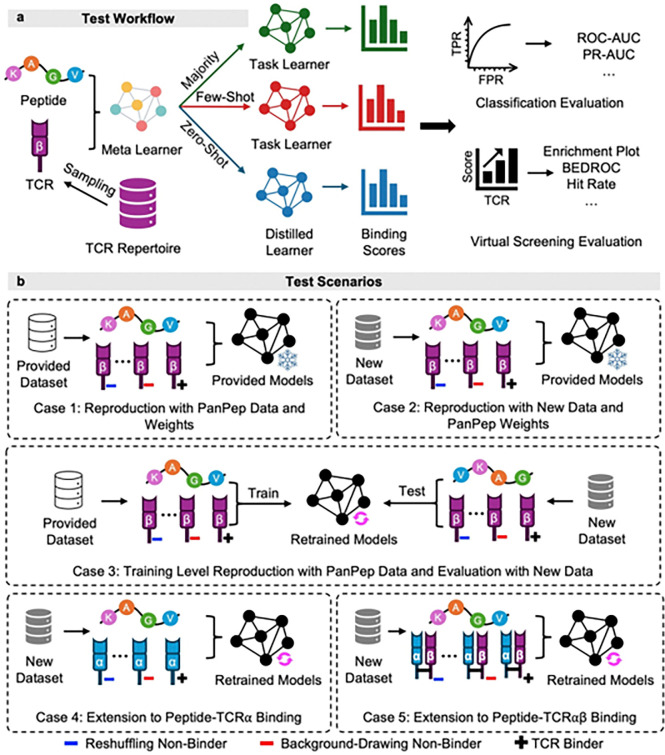
Schematic Overview of the PanPep Reusability Workflow. **(a)** Workflow for reproducing and evaluating PanPep. The model inputs the CDR3 region of the TCR chain and the target peptide. PanPep trains a meta-learner across multiple peptide datasets, which is then adapted to specific peptide tasks, yielding task learners under majority (>100 known TCR binders) and few-shot (5–100 known TCR binders) settings. For peptides with only five known TCR binders, PanPep distills a zero-shot learner from the meta-learning stage. These learners predict peptide-TCR binding scores for classification or ranking candidate TCRs for a given peptide, enabling evaluations from both classification and virtual screening perspectives. Classification performance was assessed with ROC-AUC and PR-AUC under background-drawing and reshuffling negative sampling strategies. Virtual screening performance was evaluated using Enrichment plots, BEDROC scores, and Hit rates. **(b)** Five test scenarios designed in this study to evaluate PanPep’s reusability: inference- and training-level reproducibility on the original and newly curated datasets, and extension to peptide-TCRα and peptide-TCRαβ binding recognitions.

Despite progress, predicting peptide-TCR binding remains challenging due to the extreme diversity of antigen sequences and TCR types. Models tend to overfit on peptides with abundant binders, limiting performance on rare or unseen antigens. To address this, Gao et al. proposed Pan-Peptide Meta-Learning (PanPep)^11^, a meta-learning framework designed to enhance generalization by training a meta-learner to capture shared features across diverse peptide-TCR interactions (Fig. 1a). This meta-learner serves as an adaptable base model for specific peptides through **majority learning** (more than 100 known binders) or **few-shot learning** (5 to 100 labeled binders). PanPep employs a Neural Turing Machine to support **zero-shot learning** andevaluates it using peptides with fewer than 5 annotated binders. This approach produces a tailored predictor for each antigen, offering greater adaptability to biological diversity than a single general model applied across all antigens.

While PanPep marks a significant advancement, its real-world applicability remains insufficiently evaluated. First, its classification-based assessments rely on negative sampling strategies that can introduce evaluation bias. In this field, two main negative sampling strategies were adopted (Fig. 1c), including reshuffling known positive pairs (named **reshuffling**) and randomly drawing from a background repertoire (named **background-drawing**)^14^. To avoid the false negatives that reshuffling can introduce from cross-reactive TCRs, PanPep chose background-drawing. However, this approach may lead to overestimation with antigen-irrelevant negatives^15^. Second, many tools in this domain are optimized for narrow settings, such as known peptides or common MHC alleles, and their performance often declines on novel peptides or unfamiliar TCR repertoires. Third, PanPep models only the TCRβ chain, omitting the TCRα chain due to limited data availability, which may reduce prediction confidence in clinical and immunological settings. Therefore, while PanPep represents meaningful progress, broader validation that reveals necessary methodological refinements is essential to establish the reliability and utility of TCR binder predictions in real-world immunological applications.

This report presents a comprehensive, unbiased evaluation of peptide-TCR binding predictors, focusing on PanPep and its reported competitors. We benchmarked their performance using both classification metrics under multi-fold cross-validation and enrichment-based metrics from a virtual screening perspective, which ranks candidate TCRs from an entire repertoire by their predicted binding likelihood to a given peptide, thereby minimizing biases introduced by negative sampling subsets. Leveraging newly accumulated data since PanPep’s publication, we conducted direct comparisons on an independent dataset. Furthermore, we extended PanPep to TCRα and paired TCRαβ inputs (Fig.1).

## Results

We report below the reusability testing results across five scenarios. These evaluations first examined PanPep’s inference- and training-level reproducibility using both the original dataset and a newly curated independent dataset. We then assessed its extendibility to peptide-TCRα and peptide-TCRαβ binding recognition, applying the same source code to these new task datasets.

### Inference-Level Reproducibility

#### Case 1: Reported Performance Reproductions with the PanPep-Provided Dataset

**Figure 2:**
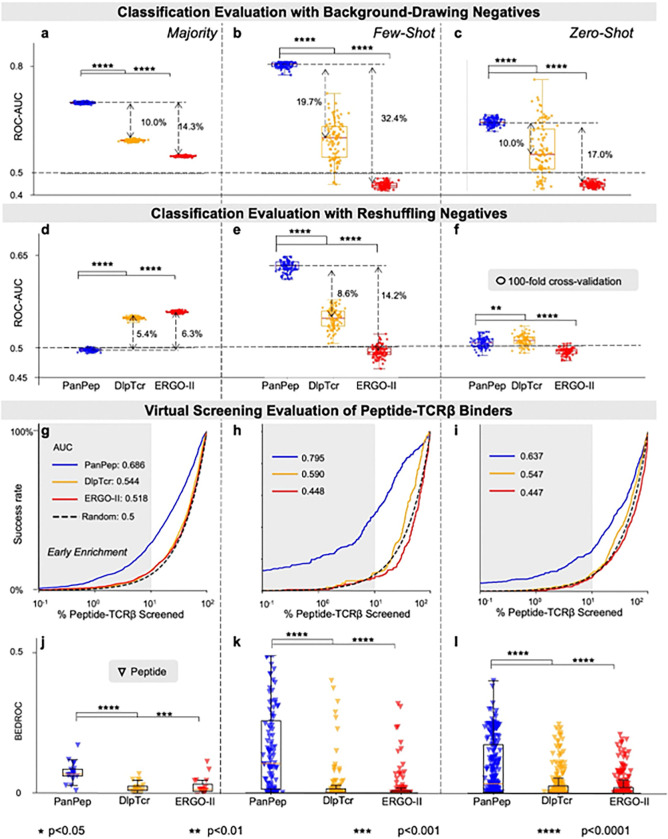
Performance Comparisons of PanPep, DlpTCR, and ERGO-II on Peptide-TCRβ Binding Data provided by PanPep. **(a-c)** ROC-AUCs from balanced classification evaluations using background-drawing negatives under 100-fold cross-validation for the majority, few-shot, and zero-shot settings. (**d-f**) ROC-AUCs from balanced classification evaluations using reshuffling negatives under 100-fold cross-validation. (**g-i**) Enrichment plots assessing virtual screening performance. The early enrichment region highlights the model’s ability to efficiently identify TCR binders with minimal experimental effort. (**j-l**) BEDROC scores quantifying early enrichment performance from a virtual screening perspective.

We assessed inference-level reproducibility by directly evaluating the original released model weights on the provided test dataset. To assess stability, we followed PanPep’s balanced classification protocol and conducted 100-fold cross-validation under two negative sampling strategies. Across the majority (peptides with ≥100 TCRβ binders), few-shot (5–100 binders), and zero-shot (<5 binders) groups (Figs. 2a–f, **Extended Data Figs. 1a-f**), boxplots of ROC-AUC and PR-AUC closely matched PanPep’s reported performance. Notably, the results for DlpTCR and ERGO-II differed from those in PanPep’s report, since these tools were benchmarked initially on separate test datasets (**Extended Data Fig. 2**). In these settings, PanPep underperformed in the majority group, and the relative performance trends among the tools were inconsistent across the two datasets. In our head-to-head comparison on PanPep’s own test set, however, PanPep outperformed both tools across all three groups under the background-drawing strategy (Figs. 2a–c, **Extended Data Figs.1a-c**). Under the reshuffling strategy (Figs. 2d–f, **Extended Data Figs. 1d-f**), its predictive power declined, approaching random guessing as previously reported^15^.

To further dissect the performance gap between the two negative sampling strategies, we decomposed PanPep’s predictions from a representative fold of the 100 -fold classification evaluations in both the majority and few-shot groups into confusion matrices (**Extended Data Fig. 3**). The results revealed that the performance drop under the reshuffling strategy was primarily attributable to an inflated false-positive rate. In this setting, negatives were generated by permuting positive peptide-TCR pairs, which introduced “hard negatives” that retained strong sequence-level or contextual similarity to true binders. PanPep misclassified these samples, likely due to over-memorizing TCRs paired with positive labels during the meta-learning training. By contrast, PanPep adopted a background-drawing strategy that produced negatives from a vastly heterogeneous TCR library, where the probability of encountering such challenging TCRs was extremely low. As a result, adaptation failed to expose the model to sufficient representative negatives to correct for this memorization bias. This indicates that (1) PanPep’s learning is strongly driven by TCR information, and (2) current background-drawing negatives poorly approximate the true distribution of non-binding TCRs, leaving a distributional gap between these two negative sampling strategies.

To extend PanPep’s analysis, we quantified performance variance across two negative sampling strategies using 100-fold cross-validation (Figs. 2a–2f, **Extend Data Figs. 1a-1f**). Variance in ROC-AUC and PR-AUC was notably higher in the few-shot and zero-shot groups (4.7–4.3% for ROC-AUC in Figs. 2bcef, 5.6–5.0% for PR-AUC in **Extend Data Figs. 1bcef**) than in the majority group (Figs. 2ad and **Extend Data Figs. 1ad**). This instability revealed that the small, artificially balanced negative subsets used in current benchmarking protocols failed to capture the scale and diversity of real peptide-TCRβ interactions, thereby introducing evaluation bias.

#### Alternative Evaluation Scheme from a Virtual Screening Perspective

In practical applications, the goal of peptide-TCRβ binding recognition is to prioritize potential TCRβ binders from a repertoire for a given peptide, thereby supporting downstream vaccine or therapeutic design. To align with this objective, we propose benchmarking peptide-TCRβ predictors using a virtual screening formulation, which more closely reflects real-world applications than either the reshuffling or background-drawing strategies (Methods). This benchmarking strategy evaluates all possible pairings between a given peptide and the entire TCRβ repertoire, especially focusing on the early enrichment of true bound pairs at top ranks. In the Enrichment plots (Figs. 2g–i), comparing with PanPep, DlpTCR, and ERGO-II, PanPep demonstrated higher success rates at the top ranks in the majority, few-shot, and zero-shot settings. This confirms PanPep’s stronger discerning power to identify TCRβ binders for a given peptide than the control tools, despite its limitations in zero-shot classification with reshuffling negatives. The Boltzmann Enhanced Discrimination of Receiver Operating Characteristic (BEDROC, see Methods) (Figs. 2j–I), which weighs early ranks, along with Hit rates (**Extended Data Figs. 1g-i**), consistently shows an advantage over the control tools.

#### Case 2: Challenging PanPep’s Generalization with a New Independent Dataset

**Figure 3:**
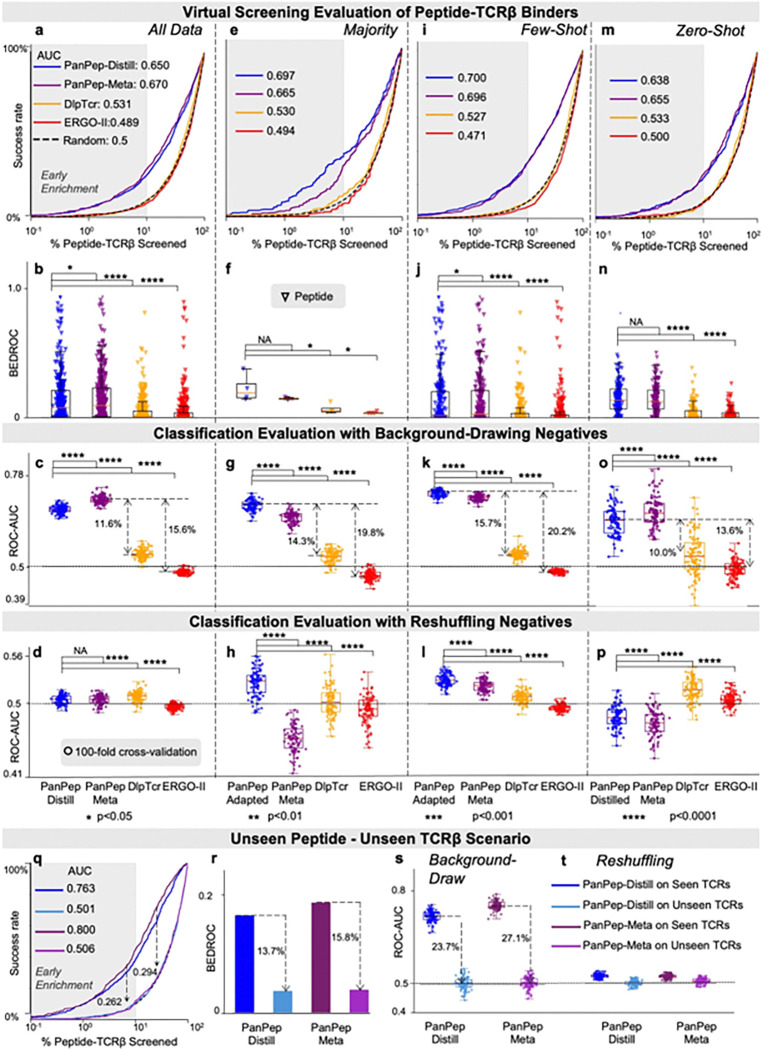
Performance of PanPep, DlpTCR, and ERGO-II in Virtual Screening and Classification Evaluations on a Newly Curated Independent Dataset. **(a-b)** PanPep’s zero-shot virtual screening evaluation on the entire dataset. **(c-d)** ROC-AUC scores from zero-shot balanced classification using background-drawing and reshuffling negatives under 100-fold cross-validation. **(e-h)** Virtual screening (enrichment plot and BEDROC) and classification (ROC-AUCs from both negative sampling strategies) evaluations under 100-fold cross-validation in the majority setting. **(i-l)** Corresponding evaluations in the few-shot setting. **(m-p)** Corresponding evaluations in the zero-shot setting. Dashed arrows indicate significant performance gaps between PanPep and controls. (**q-r**) Virtual screening performance comparison between the unseen peptide-seen TCRβ subset and the unseen peptide-unseen TCRβ subset using Enrichment plots and BEDROC scores. **(s-t)** ROC-AUCs of these two subsets from both negative sampling strategies under 100-fold cross-validation. Dashed arrows show performance gaps between the two subsets.

To evaluate PanPep’s generalizability, we constructed an independent test set containing both unseen peptides and novel TCRβ sequences that are not present in its original training data (Methods). Since this dataset was also unseen by DlpTCR and ERGO-II, we first performed a fair zero-shot comparison across all tools, including PanPep’s meta-learner and distilled versions. Enrichment plots, BEDROC scores and Hit rates (Figs. 3ab, **Extended Data Fig. 4a**) show that PanPep-meta and PanPep-distill ranked more ground-truth TCRβ binders at the top than the two control tools, underscoring the strength of its meta-training in zero-shot scenarios. Classification metrics from 100-fold cross-validation (Figs. 3cd, **Extended Data Figs. 4bc**) further support PanPep’s superior generalization. However, in the top 1% of the ranked compounds (57,099,6 compounds), less than 10% of known TCRβ binders were recovered, suggesting that its performance remained inadequate for practical deployment.

In the majority group (Figs. 3e–h, **Extended Data Figs. 4d-f**), PanPep’s task-adapted models outperformed both control tools, with significant gains in BEDROC (p<0.05), ROC-AUC (p<0.0001), and PR-AUC (p<0.001). A similar trend was observed in the few-shot setting, with consistent improvements across all metrics (Figs. 3i–I, **Extended Data Figs. 4h-j**). Adapted models significantly outperformed their meta-learners (p < 0.001) particularly in the majority group, benefiting from a larger number of support examples (> 100 for majority vs. 3~5 for few-shot) and more training iterations (1000 for majority vs. 3 for few-shot). The superiority of task adaptation was further supported by comparing zero-shot performance on the entire dataset (Figs. 3a–d) with the adapted models in the majority and few-shot settings (Figs. 3e–I). In the zero-shot group (Figs. 3m–p, and **Extended Data Figs. 4k-m**), PanPep achieved strong performance in both virtual screening and classification with background-drawing negatives. In contrast, under the reshuffling strategy (Figs.3dhip), PanPep performed nearly at random, and was underperformed by control tools trained with reshuffling negatives. These results again highlight the distributional gap between the two negative sampling strategies and its substantial impact on model performance.

To probe the source of PanPep’s generalization, we split the independent dataset into two subsets: (1) unseen peptides paired with TCRβ s from PanPep’s repertoire (i.e., these TCRβ s were present in the training data of PanPep), and (2) unseen peptides paired with novel TCRβ s not present in PanPep’s repertoire (unknown-unknown case), which forms a stricter test of generalization to truly novel combinations^15^. In this more challenging scenario, PanPep’s performance declined significantly across both classification and virtual screening metrics, as did its meta-learner (Figs. 3q–t). This gap suggests that PanPep’s success depends heavily on prior learned TCRβ repertoire.

### Training-Level Reproducibility

#### Case 3: Retrained PanPep and Evaluated on the Independent Dataset

We tested training-level reproducibility by retraining its model weights using 10-fold cross-validation on peptides from its original training set, then evaluating whether the retrained model consistently achieved similar performance to the original. This process resulted in ten independently trained PanPep models, each evaluated on the same independent testing dataset (Methods). To ensure fair comparison, we first assessed these models in zero-shot settings, alongside the two control tools.

**Figure 4:**
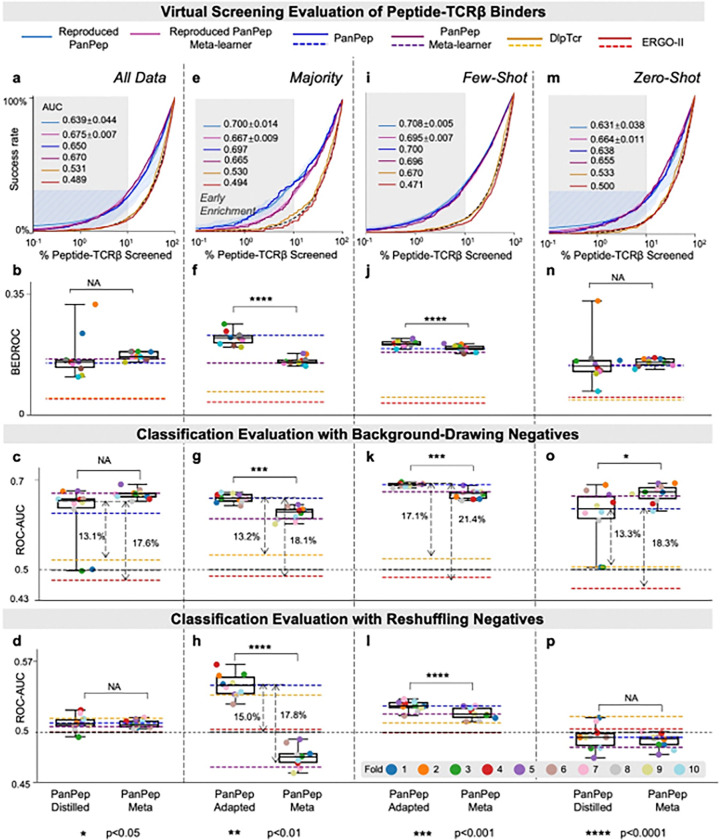
Performance of Reproduced PanPep on the Independent Dataset. **(a-d)** Enrichment plot, BEDROC scores, and ROC-AUC scores from zero-shot balanced classification evaluations using two negative sampling strategies on the entire dataset, over 10 independent PanPep reproductions. (**e-h**) Virtual screening (enrichment plots and BEDROC) and classification (ROC-AUCs from both negative sampling strategies) evaluations for the majority group in the majority setting across 10 reproductions. (**i-l**) Corresponding evaluations for the few-shot group in the few-shot setting. (**m-p**) Corresponding evaluations for the zero-shot group in the zero-shot setting. Dashed arrows indicate significant performance gaps between PanPep and controls.

In the virtual screening evaluation (Figs. 4ab, **Extended Data Fig. 5a**), PanPep maintained superior performance across metrics, including early Success rates, Hit rates, and BEDROC scores. Classification results (Figs. 4cd, **Extended Data Figs. 5bc**) showed that all ten reproduced models consistently outperformed DLCTCR and ERGO-II, with 5.6–21.4% improvements in ROC-AUC and PR-AUC. While the performance of the original PanPep model fell within the range of the ten reproduced models, we observed considerable variance (1.4–21.6%) across them. This indicates that although PanPep is reproducible, its performance is sensitive to training data splits. Furthermore, across the ten reproductions, PanPep-meta consistently outperformed PanPep-distill. These results suggest that the distillation process in PanPep may require further refinement to fully realize its potential in reproducible applications.

The ten reproduced PanPep models consistently outperformed the control tools in Success rates, Hit rates and BEDROC scores across the majority, few-shot, and zero-shot groups (Figs. 4eimfjn, **Extended Data Figs. 5dgj**). However, their classification performance (Figs. 4gkohlp, **Extended Data Figs. 5efhikl**) declined under the reshuffling negative strategy, particularly in the zero-shot setting. This suggests that PanPep’s reliance on background-drawing negatives during training may reduce robustness when facing more challenging negative samples. Applying task adaptation, especially in the majority and few-shot settings, could improve performance (Figs. 4gkhl, **Extended Data Figs. 5efhi**).

### Reusability in Peptide-TCRα Binding Recognition

#### Case 4: Extended PanPep to Peptide-TCRα Binding

The TCRα chain aids peptide-MHC recognition but is insufficient for strong binding^16^. Limited public TCRα data make peptide-TCRα binding a small-data challenge for testing PanPep’s extendibility. We derived a PanPep-TCRα dataset from DlpTcr and ERGO-II studies (Methods), excluding peptides with fewer than three binders to meet PanPep’s meta-training requirements. We conducted 10-fold cross-validation using varied sampling for PanPep, benchmarking only against DlpTcr, as ERGO-II does not support TCRα prediction.

**Figure 5:**
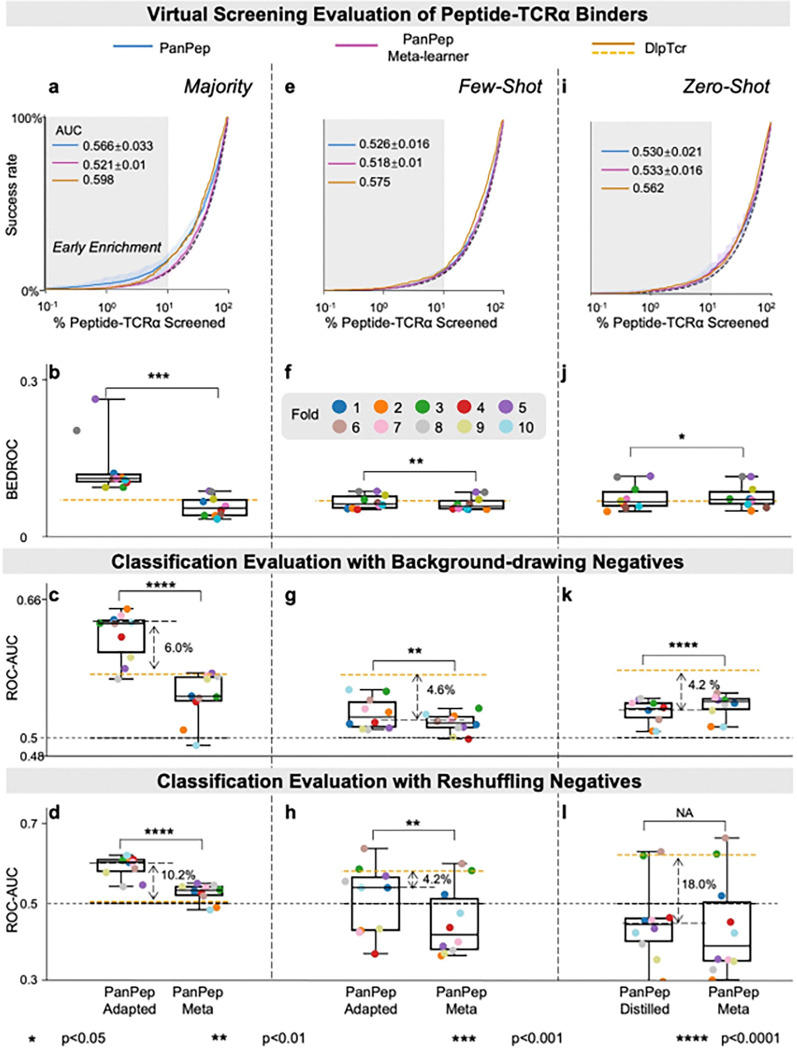
Performance of Reproduced PanPep on Peptide-TCRα Binding Scenario. **(a-d)** Enrichment plots, BEDROC scores, and ROC-AUCs from balanced classification using two negative sampling strategies, comparing PanPep’s task-adapted model with its meta-learner for the majority group in the majority setting across 10 reproductions. (**e-h**) Corresponding evaluations for the few-shot group in the few-shot setting. (**i-l**) Evaluations comparing PanPep’s distilled model with its meta-learner for the zero-shot group in the zero-shot setting. Dashed arrows indicate significant performance gaps between PanPep and controls.

In the majority group (Figs. 5a–d, **Extended Data Figs. 6a-c**), PanPep’s task-adapted models were competitive with DlpTCR in Enrichment plots, Hit rates, and BEDROC scores. ROC-AUC and PR-AUC results from balanced classification evaluations showed similar trends. Notably, under the reshuffling negative strategy, PanPep retained predictive power, while DlpTCR struggled. In the few-shot group (Figs. 5e–h, **Extended Data Figs. 6d-f**), PanPep showed limited advantage, with only 4 out of 10 adapted models outperforming DlpTCR in BEDROC. In the zero-shot setting, both models achieved comparable enrichment, though PanPep lagged in classification metrics (Figs. 5i–I, **Extended Data Figs. 6h-j**). This may be due to the smaller quantity and less diversity of PanPep’s training data (156 peptides with fewer than three TCRα binders were excluded to satisfy PanPep’s meta-training requirements). Additionally, performance variance among the 10 models was high (2.7%–36% in Fig. 5 and **Extended Data Fig. 6**), suggesting that data scarcity also hinders model robustness in peptide-TCRα prediction.

### Reusability in Peptide-TCRαβ Binding Recognition

#### Case 5: Extended PanPep to Peptide-TCRαβ Binding

The CDR3α and CDR3β loops together form the functional interface for peptide recognition and stable binding to peptide-MHC (pMHC) complexes^16^. However, most public datasets provide only TCRβ due to easier sequencing, complicating peptide-TCRαβ binding prediction. A practical workaround is to combine separate peptide-TCRα and peptide-TCRβ predictors to infer peptide-TCRαβ interactions^8^. To test PanPep’s reusability in a realistic biological context, we paired the reproduced 10-fold PanPep models trained on TCRα and TCRβ data, creating ten predictors for peptide-TCRαβ binding (Methods).

**Figure 6:**
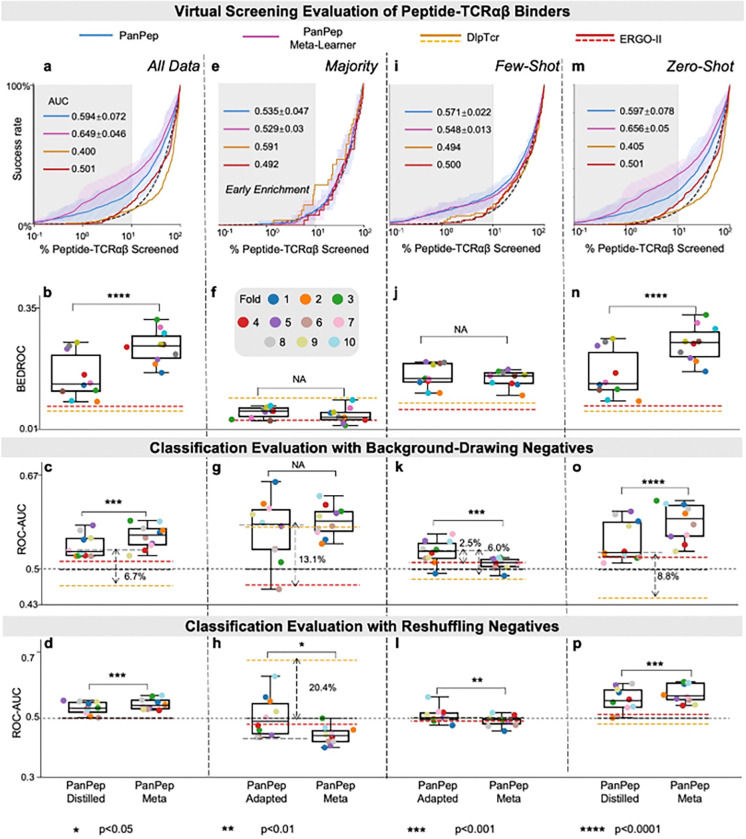
Performance of Reproduced PanPep on Peptide-TCRαβ Binding Scenario. **(a-d)** Enrichment plot, BEDROC scores, and ROC-AUC scores from balanced classification evaluations using two negative sampling strategies, comparing PanPep’s distilled model with its meta-learner, on the entire dataset in the zero-shot setting across 10 reproductions. (**e-h**) Corresponding evaluations comparing PanPep’s task-adapted model with its meta-learner for the majority group in the majority setting. (**i-l**) Corresponding evaluations for the few-shot group in the few-shot setting. (**m-p**) Corresponding evaluations for the zero-shot group in the zero-shot setting. Dashed arrows indicate significant performance gaps between PanPep and controls.

Following the DIpTCR and ERGO-II protocols, we applied these models on a peptide-TCRαβ test set derived from their studies in a zero-shot setting. The Enrichment plots, BEDROCs and Hit rates (Fig. 6ab, **Extended Data Fig. 7a**) show that PanPep’s 10-fold models consistently outperformed DIpTCR and ERGO-II, further validating PanPep’s superior extendibility and reusability. Notably, PanPep-meta again outperformed the PanPep-distill models (p-value < 0.0001 in Fig. 6b). This trend was also reflected in the 10-fold classification evaluations (p-value < 0.001 in Figs. 6cd and **Extended Data Figs. 7bc**). We further conducted task adaptation to the majority and few-shot groups using their peptide-TCRαβ support data. Only one peptide belonged to the majority group, where DlpTCR outperformed both PanPep and ERGO-II in virtual screening metrics (Figs. 6ef, **Extended Data Fig. 7d**). Few PanPep models surpassed ERGO-II after task adaptation, reflecting coordination challenges across TCRα and TCRβ models, also evident in classification results (Figs. 6gh, **Extended Data Figs. 7ef**). In the few-shot group, most PanPep models outperformed control tools, though task adaptation yielded inconsistent gains with notable variance (Figs. 6i–I, **Extended Data Figs. 7g-i**). In the zero-shot group, PanPep retained an advantage, but PanPep-distill still underperformed relative to PanPep-meta (Figs. 6m–p, **Extended Data Figs. 7j-I**). However, PanPep’s ~24% early Success rates and ~0.55 ROC-AUCs/PR-AUCs indicate that peptide-TCRαβ binding prediction remains an unsolved challenge in real-world biological contexts.

## Discussion

In this study, we demonstrated that the reported performance of PanPep can be reproduced using the provided model weights, data, and training protocol. In addition to classification evaluation, we comprehensively assessed its performance in a virtual screening setting. Compared to two control tools, PanPep showed clear advantages in its meta-learner, few-shot, and zero-shot settings, especially on an independent dataset consisting of newly released antigens and their TCRβ binders. This confirmed its generalizability to unseen antigens with few or no known TCR binders, which remains a bottleneck in the field. Beyond reproduction, we successfully reused PanPep’s code to build predictors for peptide-TCRα, and peptide-TCRαβ binding, extending its scope to more physiologically relevant contexts. These results highlight PanPep’s progress in antigen-TCR interaction modeling.

This study also revealed several limitations in PanPep’s current design. First, PanPep demonstrated limited early enrichment of TCR binders (e.g., within the top 0.1% of our VIRTUAL SCREENING evaluations), indicating the persistent challenges in real-world antigen-TCR screening. Second, the high variance observed across cross-validations suggests that the imbalance between TCR binders and non-binders remains a significant issue. Third, the marked performance decline on unseen peptide-unseen TCRβ combinations further indicated PanPep’s limited generalizability to novel TCRs. Moreover, PanPep’s few-shot adaptation and zero-shot distillation did not consistently outperform the meta-learner, implying that pre-learned knowledge may be degraded during fine-tuning, a phenomenon known as catastrophic forgetting^17^. While PanPep aimed to relate unseen antigens to learned tasks and create a zero-shot predictor using a distilled Neural Turing Machine, achieving this goal requires a universal antigen representation strategy and power-conserved distillation. In its current form, PanPep distilled all task-specific models into just three virtual representations, which may not sufficiently capture the diversity of the task space, and thus limit adaptability to novel tasks.

A promising future direction involves adopting scaling laws^18^ from molecular foundation models^19^, which improve the sequential contextual representation of amino acids by increasing model and data size via unsupervised learning strategies like masked language modeling^20^ or autoregressive modeling^21^. Unlike meta-learning, these models do not rely on task partitions and can facilitate broader generalization. Representations of antigens and peptides derived from large-scale corpora may offer more robust support for meta-learning and zero-shot task modeling. Additionally, techniques such as Elastic Weight Consolidation^17^ and Parameter-Efficient Fine-Tuning^22,23^ may provide mechanisms to preserve generalization and mitigate catastrophic forgetting during adaptation. We also recommend developing negative sampling strategies that combine broad repertoire coverage with the inclusion of representative reshuffled peptide-TCR pairs, while excluding cross-reactive cases. Such strategies would help regularize model training, mitigate overfitting to peptide- or TCR-specific features, and ultimately enhance the robustness of meta-learning and task adaptation.

This study provides a comprehensive evaluation of PanPep’s reusability in virtual screening, and its extension to TCRα and TCRαβ prediction tasks, revealing both its strengths and limitations. Our optimized implementation of PanPep supports multi-GPU parallelization to accelerate modeling and inference on the full TCR repertoire. This work also lays the foundation for evaluating future antigen-TCR binding predictors, as well as related models such as those for HLA-antigen, HLA-antigen-TCR, and protein-protein interactions.

## Methods

### Reproducibility Test Setup

In the reproducibility test, we followed PanPep’s classification evaluation protocol and additionally conducted a virtual screening evaluation. For classification, we applied PanPep’s balanced sampling strategy by selecting an equal number of unbound and bound TCRs for each peptide to construct the test set. PanPep and the control tools were evaluated on these balanced subsets. To ensure robustness, we performed 100-fold cross-validation, which allowed broader coverage of negative pairs. Performance was assessed using ROC-AUC and PR-AUC, consistent with PanPep’s original report.

The virtual screening evaluation offers a more comprehensive assessment by testing a model’s ability to achieve early enrichment. Unlike PanPep’s classification evaluation, which relies on balanced subsets, virtual screening considers all possible peptide-TCR pairs, thereby minimizing bias from subsampling. Early enrichment reflects how well a model ranks known true binders near the top of the list, which is essential for improving experimental efficiency. To assess this, we report the Enrichment plot, Hit rate, and BEDROC^24^. An Enrichment plot visualizes how effectively a model ranks true binders at the top of a sorted list of candidate TCRs for a given peptide. Candidate TCRs from an entire repertoire are sorted by predicted binding likelihood, and the cumulative proportion of true binders recovered is plotted against the proportion of the ranked list examined. Hit rate typically refers to the proportion of true TCR binders retrieved in the top-k predictions. BEDROC applies exponentially greater weight to true binders that appear at earlier ranks. Its formula is defined as follows:

(1) $${\text{BEDROC}}_{\alpha }=\frac{\alpha }{1-e^{-\alpha }}\cdot \frac{1}{n}\sum_{i=1}^{n}\;e^{-\alpha R_{i}}$$

Where n is the number of TCR binders and N is the total number of screened TCRs. The term $R_{i}=\frac{r_{i}-1}{N-1}$ normalizes the rank $r_{i}$ of the $i^{\text{th}}$ TCR binder. The parameter α controls the emphasis on early top ranks, with a common choice in our work α=20 placing approximately ~80% of the weight on the top 1% ranks.

The virtual screening evaluation required extensive computation due to the large number of peptide-TCR pairs. To accelerate this process, we optimized PanPep’s code to support multi-GPU parallelism. All peptides were divided into k groups, where k equals the number of available GPUs, and each GPU processed one group. For each peptide, its corresponding TCR pairs were batched and processed on the assigned GPU. This parallelized workflow was executed on a machine with 8 GPUs, a 56-core CPU, and 512 GB of physical memory, using a batch size of 150 . Virtual screening metrics were implemented using the cuML Python package to leverage GPU-based matrix operations. These improvements enabled efficient and timely execution of the virtual screening evaluations.

### Training and Test Data Provided by PanPep

In the inference-level reusability evaluation, we utilized PanPep’s original test dataset, which includes 276 peptides and their 34,711 TCRβ binders, forming a total of 36,487 peptide-TCRβ binding pairs. The dataset was categorized into majority, few-shot, and zero-shot groups, comprising 25,122, and 129 peptides, respectively. For the balanced classification evaluation, an equal number of non-binding TCRβ sequences were either randomly sampled from a background repertoire of 57,107,565 TCRβ sequences or generated by reshuffling the 34,711 known binders, corresponding to different negative sampling strategies. In the virtual screening evaluation, each peptide was tested against the entire background repertoire of 57,107,565 TCRβ sequences to identify and rank the most likely binding candidates.

For the training-level reusability evaluation, PanPep’s original training dataset was divided into 10 folds, each containing 188 peptides with varying proportions of majority, few-shot, and zero-shot samples. These folds were used to retrain PanPep’s meta-learner under 10-fold cross-validation. During training, balanced negative sampling was applied by selecting non-binding TCRβ sequences from the same background library for each peptide, consistent with PanPep’s original negative sampling protocol. The resulting 10 meta-learner models were then evaluated on PanPep’s original test dataset using both classification and virtual screening metrics.

### Independent Testing Data Construction

We followed PanPep’s data curation protocol to retrieve all available human HLA class I-related peptide and TCRβ binding records from the IEDB^25^, VDJdb^26^, and McPAS^27^ TCR databases, excluding the PIRD database due to its recent inaccessibility. PanPep’s data quality-control criteria were then applied to remove low-confidence records. After excluding PanPep’s original training and evaluation data, the remaining records were used as the positive set for our independently curated benchmark dataset. This dataset includes 670 unique peptides and 4,362 unique TCRβ sequences, forming 4,377 peptide-TCRβ binding pairs. Following PanPep’s task definitions, these peptides were grouped into majority, few-shot, and zero-shot categories, containing 4,150 , and 516 peptides, respectively. The corresponding non-binding TCRβ set was constructed either by randomly sampling from PanPep’s control repertoire of 57,107,565 TCR sequences or by reshuffling the 4,362 known binders, depending on the chosen negative sampling strategy.

After gathering all data sources, we identified 11,550 novel TCRβ sequences that were not present in PanPep’s original TCRβ repertoire. This allowed us to construct an unseen peptide and unseen TCRβ subset to evaluate PanPep’s reasoning ability in completely novel settings. Specifically, these 11,550 TCRβ s were treated as an unseen TCRβ library, and 391 unseen peptides known to bind them led to 1,991 peptide-TCRβ binding pairs. All non-binding pairs between these peptides and the unseen TCRβ library were used as negative samples in this subset.

### Construction of Peptide-TCRα and TCRαβ Binding Datasets for Reusability Test

To extend PanPep for peptide-TCRα binding prediction, we applied its meta-training framework using DlpTCR’s training data^7^, which included 273 unique peptides and 4,508 unique TCRα sequences forming 4,922 binding pairs. A total of 156 peptides with fewer than three TCRα binders were excluded, as they did not meet the minimum support and query requirements for PanPep’s meta-training protocol. For evaluation, we compiled peptide-TCRα binding records from IEDB, VDJdb, and McPAS databases, together with the test data from the DIpTCR and ERGO-II studies. Both PanPep and DIpTCR were evaluated on this compiled test set (ERGO-II does not support peptide-TCRα binding prediction), which contained 215 unique peptides and 1,126 unique TCRα sequences with 14,436 binding pairs. Following PanPep’s task definitions, the test set was partitioned into 11 majority, 186 few-shot, and 931 zero-shot tasks. All TCRα sequences from both the training and testing data were pooled into a TCRα library of 37,461 sequences. Negatives were constructed either by pairing peptides with sequences from this library or by reshuffling the known 1,126 binders, depending on the adopted sampling strategy.

Similarly, we compiled peptide-TCRαβ binding records from IEDB, VDJdb, and McPAS, together with data from the DIpTCR and ERGO-II studies, yielding 286 unique peptides and 472 unique TCRαβ sequences with 723 documented interactions. Due to the limited availability of peptide-TCRαβ data, all records were reserved exclusively for benchmarking. This dataset was categorized into 1 majority, 18 few-shot, and 267 zero-shot tasks. All TCRαβ sequences were consolidated into a library of 24,191 sequences. Negatives were generated either by pairing the peptides with sequences from this library or by reshuffling the 472 known binding pairs, depending on the adopted sampling strategy.

### Extending PanPep to Peptide-TCRα and TCRαβ Binding Recognition

We used the training code provided by PanPep’s authors to perform meta-learning on the peptide-TCRα dataset, modifying the input to accept a peptide sequence and the corresponding CDR3α sequence of a TCR. The resulting TCRα-oriented meta-learner was fine-tuned using task-specific support data for the majority and few-shot settings. For the zero-shot setting, peptide-TCRα models were generated by distilling task learners from the meta-learning process, following PanPep’s zero-shot protocol. To evaluate the model’s stability, we conducted 10-fold cross-validation throughout the peptide-TCRα binding modeling process.

For peptide-TCRαβ binding prediction, the CDR3a and CDR3 β sequences were input separately into PanPep-TCRα and PanPep-TCRβ models, along with the peptide sequence. The individual predictions from each model were averaged to generate a final binding score for the peptide-TCRαβ pair. For majority and few-shot tasks, the meta-learners trained on peptide-TCRα and peptide-TCRβ data were fine-tuned independently using their respective support sets. In the zero-shot setting, the distilled PanPep-TCRα and PanPep-TCRβ models were applied directly without further adaptation. To assess the overall stability of this approach, we also evaluated peptide-TCRαβ binding performance using the 10-fold cross-validated PanPep-TCRα and PanPep-TCRβ models.

## Supplementary Material

Supplementary Files

This is a list of supplementary fi les associated with this preprint. Click to download.

• ExtendedData.docx

## Data Availability

The dataset used by PanPep is publicly available on Zenodo (https://doi.org/10.5281/zenodo.7544387), and our newly curated dataset has been deposited on Zenodo as well (https://doi.org/10.5281/zenodo.16943691).

## Code Availability

The original code of PanPep is available at https://github.com/bm2-lab/PanPep. Our code to run the reproducibility results and to analyze the reusability is available via GitHub at https://github.com/coffee19850519/PanPep_Reusability.
